# Response to First-Line Ritonavir-Boosted Protease Inhibitors (PI/r)-Based Regimens in HIV Positive Patients Presenting to Care with Low CD4 Counts: Data from the Icona Foundation Cohort

**DOI:** 10.1371/journal.pone.0156360

**Published:** 2016-06-27

**Authors:** Antonella d’Arminio Monforte, Alessandro Cozzi-Lepri, Franco Maggiolo, Giuliano Rizzardini, Paolo Emilio Manconi, Nicola Gianotti, Tiziana Quirino, Carmela Pinnetti, Stefano Rusconi, Andrea De Luca, Andrea Antinori, Icona Foundation Study cohort

**Affiliations:** 1 University of Milan, Department of Health Sciences, Clinic of Infectious and Tropical Diseases, ASST Santi Paolo e Carlo, Milan, Italy; 2 University College, London, United Kingdom; 3 Spedali Riuniti, Bergamo, Italy; 4 L Sacco Hospital, Milan, Italy; 5 University of Cagliari, Cagliari, Italy; 6 San Raffaele Hospital, Milan, Italy; 7 Busto Arsizio Hospital, Varese, Italy; 8 INMI L Spallanzani, Rome, Italy; 9 University of Milan, Sacco Hospital, Milan, Italy; 10 University of Siena, Siena, Italy; University of Pittsburgh, UNITED STATES

## Abstract

**Background:**

There are no data comparing the response to PI/r-based regimens in people presenting for care with low CD4 counts or AIDS (LC).

**Aim:**

To compare the response to LPV/r-, DRV/r- or ATV/r-based cART regimens in LC initiating cART from ART-naive.

**Methods:**

We included people enrolled in Icona with either CD4 counts ≤350 cells/mm^3^ (low CD4-LC) or CD4 counts ≤200 cells/mm^3^ (very low CD4-VLC) and/or AIDS, starting their first PI/r-based regimen after 2008. Initial regimens were compared by intention-to-treat: i) time to viral failure (VF) (first of 2 consecutive VL>200 copies/mL after≥6 months); II) time to PI/r discontinuation/switching for any cause (TD) and for toxicity (TDT); III) treatment failure (TF) (VF or TD). Kaplan-Meier and Cox analyses were used.

**Results:**

1,362 LC patients were included (DRV/r 607; ATV/r 552; LPV/r 203); 813 VLC. In a median of 18 months (IQR:7–35), the 1-year probability of VF and TF were 2.8% (1.9–3.8) and 21.1% (18.7–23.4). In the adjusted analysis, patients initiating ATV/r had a 53% lower chance, and those initiating DRV/r a 61% lower chance of TD, as compared to LPV/r; the risk of TF was more likely in people starting LPV/r. Results were similar among VLC; in this subgroup LPV/r including regimens demonstrated a lower chance of VF.

**Conclusions:**

We confirmed in LC a low chance of virological failure by 1 year, with small differences according to PI/r. However, larger differences were observed when comparing longer-term endpoints such as treatment failure. These results are important for people presenting late for care.

## Introduction

The recently published START trial has demonstrated that antiretroviral therapy (cART) should be started as early as possible after HIV diagnosis [1]; this is based on solid clinical evidence of what had up to this point only been shown in biological and observational studies [2–3]. Altogether these findings have dramatically changed the approach to cART and recently published guidelines are all in favour of initiation of ART as soon as possible after HIV diagnosis [4–7].

Nevertheless, worldwide and including in resource-rich countries, a rate ranging from 40 to 60% of patients are diagnosed when they have already an AIDS defining disease or a low CD4 count, and these individuals will not benefit from new indications on early therapy [8–9]. Historically, HIV-infected patients have been labelled as ‘late presenters’ for care on the basis of the established threshold described in older versions of the treatment guidelines, e.g. individuals with a diagnosis of AIDS and/or CD4 counts of less than 350 cells/mm^3^ or individuals with a even more advanced stage of HIV disease (a diagnosis of AIDS and/or CD4 counts of less than 200 cells/mm^3^) at the time of their first presentation for ´[10]. These groups have been extensively described in large collaborative cohort studies both in Europe and in other parts of the world (8, 9). At present, the definition of ‘late presenters’ is no more applicable, as everybody diagnosed with HIV should be treated independently from CD4 counts, otherwise could be defined as ‘late presenter’. We therefore choose the definitions of ‘low CD4 counts (LC) and very low CD4 count (VLC) to define people diagnosed with AIDS and/or CD4 count ≤350 cells/mm^3^ or ≤200 cells/mm^3^, respectively.

Although newer drugs belonging to the integrase inhibitors class (raltegravir, dolutegravir and elvitegravir) as well as newer generation NNRTI (rilpivirine) are now the most commonly used drugs included as third agents in first-line cART, darunavir/r and atazanavir/r are still among the preferred options in most treatment guidelines, including Italian ones. Lopinavir/r, in contrast, is now only considered as an alternative option by all Guidelines [4–7]. Nevertheless, ritonavir-boosted protease inhibitors (PI/r)-containing regimens remain regimens with strong supporting evidence of clinical efficacy, for which clinicians have long term experience in clinical use and are a still considered as first line options in persons with presumably low adherence or in cases with missing drug resistance tests before starting cART, due to their high genetic barrier [4–7].

Head to head randomised clinical trials comparing individual PI/r, lopinavir/ritonavir (LPV/r), darunavir/ritonavir (DRV/r) and atazanavir/ritonavir (ATV/r) are not numerous and none of those performed up to date could clearly demonstrate the superiority of one of these over the others with regards to potency in the subset of severely immunodepressed patients. With respect to the safety profile, LPV/r has been shown to be less tolerable than the other two and this is the main reason why it is no longer considered a preferred option [11–13]. However, the choice of the best regimen in individuals with advanced HIV disease remains particularly challenging. Randomized trials sufficiently powered to compare treatment response in people with advanced disease starting PI/r-based regimens are either lacking or suffer from small sample size or short duration of follow-up [11–13].

Even if drug comparative analyses using observational data are, by nature, controversial and conflict remains regarding whether they should performed at all, they may convey useful information when no randomized comparison has been performed or planned. Also, patients have generally been followed up for longer in the observational setting as compared to clinical trials and cohorts include populations that are often selected out of randomized studies.

For these reasons, we performed an analysis of the outcomes of severely immunodepressed patients in the ICONA cohort initiating a first cART regimen with ritonavir boosted PI-including regimens, aiming at testing whether their response to treatment may vary according to the type of PI/r initiated.

## Methods

### Patient population

ICONA Foundation Study (ICONA) is a multi-centre prospective observational study of HIV-1-infected patients, which was set up in 1997. Eligible patients are those starting cART when they are naive to antiretrovirals, regardless of the reason for which they had never been previously treated and of the stage of their disease. The ICONA Foundation study has been approved by IRB of all the participating centers; sensitive data from patients are seen only in aggregate form. All patients sign a consent form to participate in ICONA, in accordance with the ethical standards of the committee on human experimentation and the Helsinki Declaration (1983 revision). Demographic, clinical and laboratory data and information on therapy are collected for all participants and recorded using electronic data collection [www.icona.org]. Details of the study are described elsewhere [14].

We focussed on two groups of individuals using the historical definitions of late presenters as developed by an International Consensus:

1. diagnosis of AIDS and/or CD4 count ≤350 cells/mm^3^ (low CD4 count group -LC)
2. diagnosis of AIDS and/or CD4 count ≤200 cells/mm^3^ (very low CD4 count -VLC)

Within these groups, we selected individuals who initiated their initial cART regimen when there were still ART-naïve after 31/12/2008. These initial cART had to include two nuclesoside reverse transcriptase inhibitors (NRTIs, either tenofovir+emtricitabine (TDF/FTC) or abacavir+lamivudine (ABC/3TC) + either ritonavir-boosted lopinavir (LPV/r), or ritonavir-boosted atazanavir (ATV/r), or ritonavir-boosted darunavir (DRV/r). In addition, individuals had to have at least one month of clinical follow-up to be included in the analysis.

The response to these initial regimens was compared according to the specific PI/r started with respect of four main outcomes:

1. time to virological failure (VF) defined at time of the first of 2 consecutive VL>200 copies/mL after ≥6 months of ART;
2. time to treatment discontinuation/switching of the PI/r component of the regimen regardless of the reason (TD) and
3. discontinuation because of toxicity (TDT);
4. treatment failure (TF) defined as time to VF or to discontinuation/switching of the PI/r component of the regimen.

All causes of discontinuation are collected in the ICONA database as reported by the treating physician. In particular, clinicians are asked to report for each single drug which was the main reason for discontinuing it. These reasons include the broad categories of simplification (defined either as the reduction of drugs included in the regimen or the decrease in daily doses or pills), intolerance, toxicity, failure (either virological, immunological or clinical), non-adherence, planned interruption (including structured treatment interruption, end of pregnancy and medical decision) and other causes (including patients decision, pregnancy, enrolment or ending of a clinical trial and drug-drug interaction).

The main exposure of interest was the specific PI/r initiated and a separate Cox regression model was fitted for the risk of VF, TD, TDT, and TF. The same set of potential confounders were considered for all four models which included: age, gender, comorbidities, calendar year of starting cART, nadir lymphocyte T CD4+ cell count, HIV-RNA plasma level at cART initiation, whether TDF/FTC or ABC/3TC were used in the initial regimen.

### Statistical analysis

The main characteristics of the two study populations (LC and VLC) have been described and compared according to the initial PI/r started. Prevalence in the categorical variables has been compared across the strata using a chi-square test. Continuous variables have been compared using non-parametric tests.

The response to initial regimen was compared using an intention-to-treat analysis with respect to the four main outcomes described in the previous paragraph (i.e. patients were allocated to their initial PI/r regardless of subsequent treatment switches).

Standard survival analysis by Kaplan-Meier (KM) curves and Cox regression models was used; KM estimates with 95% confidence intervals at 1 and 3 years from cART initiation were computed and KM curves shown; unadjusted and adjusted relative hazards (RH) were computed and reported. Confounding was addressed by standard multivariable modelling including potential confounders in the adjusted models. Cox regression models were further stratified for clinical site of enrolment. All analyses have been performed using SAS version 9.4.

## Results

A total of 1,362 LC patients fulfilling the inclusion criteria were analysed: 607 (44.6%) received DRV/r, 552 (40.5%) ATV/r and 203 (14.9%) LPV/r-including regimens. Characteristics of LC patients according to PI-including regimen are described on Table 1: 326 (23.9%) patients were women, 25% were non-Italian, median CD4 count was 165 cells/mmc (IQR: 59–266), median HIV RNA log 10 copy levels/ml was 5.00 (4.40–5.49), 150 (11.0%) had a diagnosis of AIDS at baseline. There were wide variations in demographic and clinical variables according to regimen: LPV/r receiving regimens individuals started cART in earlier years, were more frequently women, less frequently infected through homosexual contacts, more frequently non Italians (see Table 1). Subjects starting ATV/r including regimens had less frequently a AIDS diagnosis at baseline [AIDS at baseline: 88 (14.5%) DRV/r; 26 (12.8%) LPV/r; 36(6.5%) ATV/r; p <.001]. Backbone with nucleoside pairs was mainly represented by tenofovir+emicitrabine (TDF+FTC), given in 1207 (88.6%) of patients.

**Table 1:** Characteristics of patients—LC group: CD4 ≤350 cells/mm^3^ or AIDS.

	PI/r started				
Characteristics	DRV/r	ATV/r	LPV/r	p-value*	Total
	N = 607	N = 552	N = 203		N = 1362
***Gender*, *n(%)***				0.021	
Female	134 (22.1%)	128 (23.2%)	64 (31.5%)		326 (23.9%)
***Mode of HIV Transmission*, *n(%)***				0.002	
IDU	42 (7.0%)	64 (11.7%)	22 (10.8%)		128 (9.5%)
Homosexual contacts	230 (38.2%)	182 (33.2%)	53 (26.1%)		465 (34.3%)
Heterosexual contacts	278 (45.8%)	271 (49.1%)	107 (52.7%)		656 (48.2%)
Other/Unknown	52 (8.6%)	32 (5.8%)	21 (10.3%)		105 (7.8%)
***Nationality*, *n(%)***				0.002	
Not Italian	151 (24.9%)	133 (24.1%)	57 (28.1%)		341 (25.0%)
***AIDS diagnosis*, *n(%)***				<.001	
Yes	88 (14.5%)	36 (6.5%)	26 (12.8%)		150 (11.0%)
***CVD diagnosis*, *n(%)***				0.114	
Yes	7 (1.2%)	2 (0.4%)	0 (0.0%)		9 (0.7%)
***HBsAg*, *n(%)***				0.638	
Negative	515 (84.8%)	462 (83.7%)	178 (87.7%)		1155 (84.8%)
Positive	10 (1.6%)	13 (2.4%)	4 (2.0%)		27 (2.0%)
Not tested	82 (13.5%)	77 (13.9%)	21 (10.3%)		180 (13.2%)
***HCVAb*, *n(%)***				0.115	
Negative	486 (80.1%)	434 (78.6%)	165 (81.3%)		1085 (79.7%)
Positive	52 (8.6%)	70 (12.7%)	21 (10.3%)		143 (10.5%)
Not tested	69 (11.4%)	48 (8.7%)	17 (8.4%)		134 (9.8%)
Hepatitis co-infection*, *n(%)*				0.077	
No	453 (74.6%)	393 (71.2%)	157 (77.3%)		1003 (73.6%)
Yes	62 (10.2%)	83 (15.0%)	24 (11.8%)		169 (12.4%)
Not tested	92 (15.2%)	76 (13.8%)	22 (10.8%)		190 (14.0%)
Calendar year of baseline**				<.001	
Median (IQR)	2012 (2011, 2014)	2012 (2011, 2013)	2011 (2009, 2012)		2012 (2011, 2013)
***Age*, *years***				0.182	
Median (IQR)	40 (33, 49)	39 (32, 47)	39 (33, 47)		39 (33, 47)
***CD4 count nadir*, *cells/mmc***				<.001	
Median (IQR)	131 (42, 248)	212 (99, 285)	150 (58, 246)		165 (59, 266)
***CD8 count*, *cells/mmc***				0.002	
Median (IQR)	675 (441, 1001)	739 (514, 1151)	758 (513, 1147)		708 (483, 1068)
***Viral load at first cART*, *log10 copies/mL***				0.008	
Median (IQR)	5.07 (4.43, 5.55)	4.89 (4.26, 5.42)	4.99 (4.47, 5.46)		5.00 (4.40, 5.49)
***Site geographical position*, *n(%)***				<.001	
North	310 (51.1%)	336 (60.9%)	99 (48.8%)		745 (54.7%)
Center	203 (33.4%)	174 (31.5%)	74 (36.5%)		451 (33.1%)
South	94 (15.5%)	42 (7.6%)	30 (14.8%)		166 (12.2%)
***Diabetes*, *n(%)***				0.049	
Yes	9 (1.5%)	14 (2.5%)	0 (0.0%)		23 (1.7%)
***Smoking*, *n(%)***				0.020	
No	293 (48.3%)	240 (43.5%)	103 (50.7%)		636 (46.7%)
Yes	182 (30.0%)	213 (38.6%)	61 (30.0%)		456 (33.5%)
Unknown	132 (21.7%)	99 (17.9%)	39 (19.2%)		270 (19.8%)
***Total cholesterol*, *mg/dL***				0.009	
Median (IQR)	151 (123, 180)	157 (134, 182)	160 (140, 184)		155 (131, 182)
***HDL cholesterol*, *mg/dL***				0.017	
Median (IQR)	36 (28, 44)	37 (31, 46)	37 (30, 47)		36 (30, 45)
***Use of statins*, *n(%)***				0.601	
Yes	7 (1.2%)	4 (0.7%)	1 (0.5%)		12 (0.9%)
***Use of blood pressure lowering drugs*, *n(%)***				0.819	
Yes	20 (3.3%)	21 (3.8%)	6 (3.0%)		47 (3.5%)
***Time from HIV diagnosis to date of starting cART*, *months***				<.001	
Median (IQR)	1 (1, 5)	2 (1, 25)	2 (1, 17)		2 (1, 13)
***egfr (CKD_Epi formula)*, *ml/min/1*.*73m***^***2***^				0.927	
Median (IQR)	106.7 (93.27, 117.3)	107.2 (96.61, 116.4)	107.7 (93.30, 117.8)		107.1 (94.61, 116.8)
***Blood glucose*, *mg/dL***				0.420	
Median (IQR)	86 (79, 95)	87 (80, 94)	86 (79, 94)		86 (79, 94)
***NRTI pair started*, *n(%)***				0.964	
Tenofovir/Emtricitabine	537 (88.5%)	489 (88.6%)	181 (89.2%)		1207 (88.6%)
Abacavir//Lamivudine	70 (11.5%)	63 (11.4%)	22 (10.8%)		155 (11.4%)
***DRV dosage*, *n(%)***					
BID	87 (14.3%)				
QD	489 (80.6%)				
Unknown	31 (5.1%)				
***Follow-up*, *months***				<.001	
Median (IQR)	17 (6, 32)	22 (9, 38)	15 (5, 37)		18 (7, 35)

*Chi-square or Kruskal-Wallis test as appropriate

DRV/r was given mainly as QD regimen (in 489 patients-80.6%), but 87 patients (14.3%) were given 600mg BID dosage and in 31 (5.1%) the scheduled dosage was unknown.

A total of 813 of the 1,362 patients (59.7%) fulfilled the definition of presenters with very low CD4 count (VLC); 414 (50.9%) initiated DRV/r-, 268 (33,0%) initiated ATV/r- and 131 (16.1%) initiated LPV/r-including regimens. Differences among the treatment groups are shown in Table 2 and mostly confirmed the differences occurring in the wider population of LC.

**Table 2:** Characteristics of patients—VLC group: CD4 count less than 200 or AIDS.

	PI/r started				
	DRV/r	ATV/r	LPV/r	p-value*	Total
	N = 414	N = 268	N = 131		N = 813
***Gender*, *n(%)***				0.037	
Female	89 (21.5%)	59 (22.0%)	42 (32.1%)		190 (23.4%)
***Mode of HIV Transmission*, *n(%)***				0.002	
IDU	22 (5.3%)	34 (12.8%)	12 (9.2%)		68 (8.4%)
Homosexual contacts	140 (34.0%)	71 (26.7%)	30 (22.9%)		241 (29.8%)
Heterosexual contacts	207 (50.0%)	143 (53.4%)	73 (55.7%)		423 (52.0%)
Other/Unknown	43 (10.4%)	18 (6.8%)	16 (12.2%)		77 (9.5%)
***Nationality*, *n(%)***				0.001	
Not Italian	112 (27.1%)	69 (25.7%)	42 (32.1%)		223 (27.4%)
***AIDS diagnosis*, *n(%)***				0.033	
Yes	88 (21.3%)	36 (13.4%)	26 (19.8%)		150 (18.5%)
***CVD diagnosis*, *n(%)***				0.387	
Yes	4 (1.0%)	1 (0.4%)	0 (0.0%)		5 (0.6%)
***HBsAg*, *n(%)***				0.463	
Negative	353 (85.3%)	226 (84.3%)	117 (89.3%)		696 (85.6%)
Positive	7 (1.7%)	8 (3.0%)	1 (0.8%)		16 (2.0%)
Not tested	54 (13.0%)	34 (12.7%)	13 (9.9%)		101 (12.4%)
***HCVAb*, *n(%)***				0.066	
Negative	334 (80.7%)	209 (78.0%)	109 (83.2%)		652 (80.2%)
Positive	31 (7.5%)	36 (13.4%)	10 (7.6%)		77 (9.5%)
Not tested	49 (11.8%)	23 (8.6%)	12 (9.2%)		84 (10.3%)
Hepatitis co-infection*, *n(%)*				0.020	
No	314 (75.8%)	192 (71.6%)	106 (80.9%)		612 (75.3%)
Yes	38 (9.2%)	44 (16.4%)	11 (8.4%)		93 (11.4%)
Not tested	62 (15.0%)	32 (11.9%)	14 (10.7%)		108 (13.3%)
Calendar year of baseline**				<.001	
Median (IQR)	2012 (2011, 2014)	2012 (2011, 2013)	2011 (2009, 2012)		2012 (2011, 2013)
***Age*, *years***				0.964	
Median (IQR)	41 (34, 50)	41 (34, 50)	41 (34, 49)		41 (34, 50)
***CD4 count nadir*, *cells/mmc***				0.006	
Median (IQR)	68 (29, 132)	99 (40, 153)	85 (33, 150)		78 (32, 143)
***CD8 count*, *cells/mmc***				0.107	
Median (IQR)	561 (343, 842)	609 (408, 955)	655 (370, 1047)		593 (370, 906)
***Viral load at first cART*, *log10 copies/mL***				0.397	
Median (IQR)	5.26 (4.65, 5.72)	5.18 (4.60, 5.60)	5.09 (4.57, 5.63)		5.20 (4.61, 5.67)
***Site geographical position*, *n(%)***				0.136	
North	207 (50.0%)	149 (55.6%)	61 (46.6%)		417 (51.3%)
Center	140 (33.8%)	91 (34.0%)	53 (40.5%)		284 (34.9%)
South	67 (16.2%)	28 (10.4%)	17 (13.0%)		112 (13.8%)
***Diabetes*, *n(%)***				0.084	
Yes	8 (1.9%)	9 (3.4%)	0 (0.0%)		17 (2.1%)
***Smoking*, *n(%)***				0.282	
No	201 (48.6%)	120 (44.8%)	64 (48.9%)		385 (47.4%)
Yes	116 (28.0%)	96 (35.8%)	39 (29.8%)		251 (30.9%)
Unknown	97 (23.4%)	52 (19.4%)	28 (21.4%)		177 (21.8%)
***Total cholesterol*, *mg/dL***				0.001	
Median (IQR)	143 (117, 173)	153 (130, 177)	164 (138, 183)		149 (125, 177)
***HDL cholesterol*, *mg/dL***				0.062	
Median (IQR)	33 (26, 42)	36 (29, 45)	35 (28, 43)		34 (27, 43)
***Use of statins*, *n(%)***				0.805	
Yes	5 (1.2%)	2 (0.7%)	1 (0.8%)		8 (1.0%)
***Use of blood pressure lowering drugs*, *n(%)***				0.711	
Yes	14 (3.4%)	8 (3.0%)	6 (4.6%)		28 (3.4%)
***Time from HIV diagnosis to date of starting cART*, *months***				0.527	
Median (IQR)	1 (0, 2)	1 (0, 2)	1 (0, 5)		1 (0, 2)
***egfr (CKD_Epi formula)*, *ml/min/1*.*73m***^***2***^				0.784	
Median (IQR)	108.5 (93.07, 117.8)	107.9 (95.00, 115.8)	107.7 (93.87, 117.3)		108.0 (93.82, 116.8)
***Blood glucose*, *mg/dL***				0.676	
Median (IQR)	86 (79, 96)	88 (80, 96)	87 (78, 97)		87 (79, 96)
***NRTI pair started*, *n(%)***				0.593	
Tenofovir/Emtricitabine	371 (89.6%)	246 (91.8%)	117 (89.3%)		734 (90.3%)
Abacavir//Lamivudine	43 (10.4%)	22 (8.2%)	14 (10.7%)		79 (9.7%)
***DRV dosage*, *n(%)***					
BID	61 (14.7%)				
QD	327 (79.0%)				
Unknown	26 (6.3%)				
***Follow-up*, *months***				0.076	
Median (IQR)	16 (6, 31)	17 (7, 35)	13 (5, 34)		15 (6, 33)

*Chi-square or Kruskal-Wallis test as appropriate

Over a median follow-up of 18 months (IQR:7–35), 57 LC patients (4.2%) experienced virological failure (VF), 507 (37.2%) experienced treatment discontinuation (TD), 97 (7.1%) discontinuation because of toxicity (TDT) and 485 (35.6%) treatment failure (TF).

**Fig 1:**
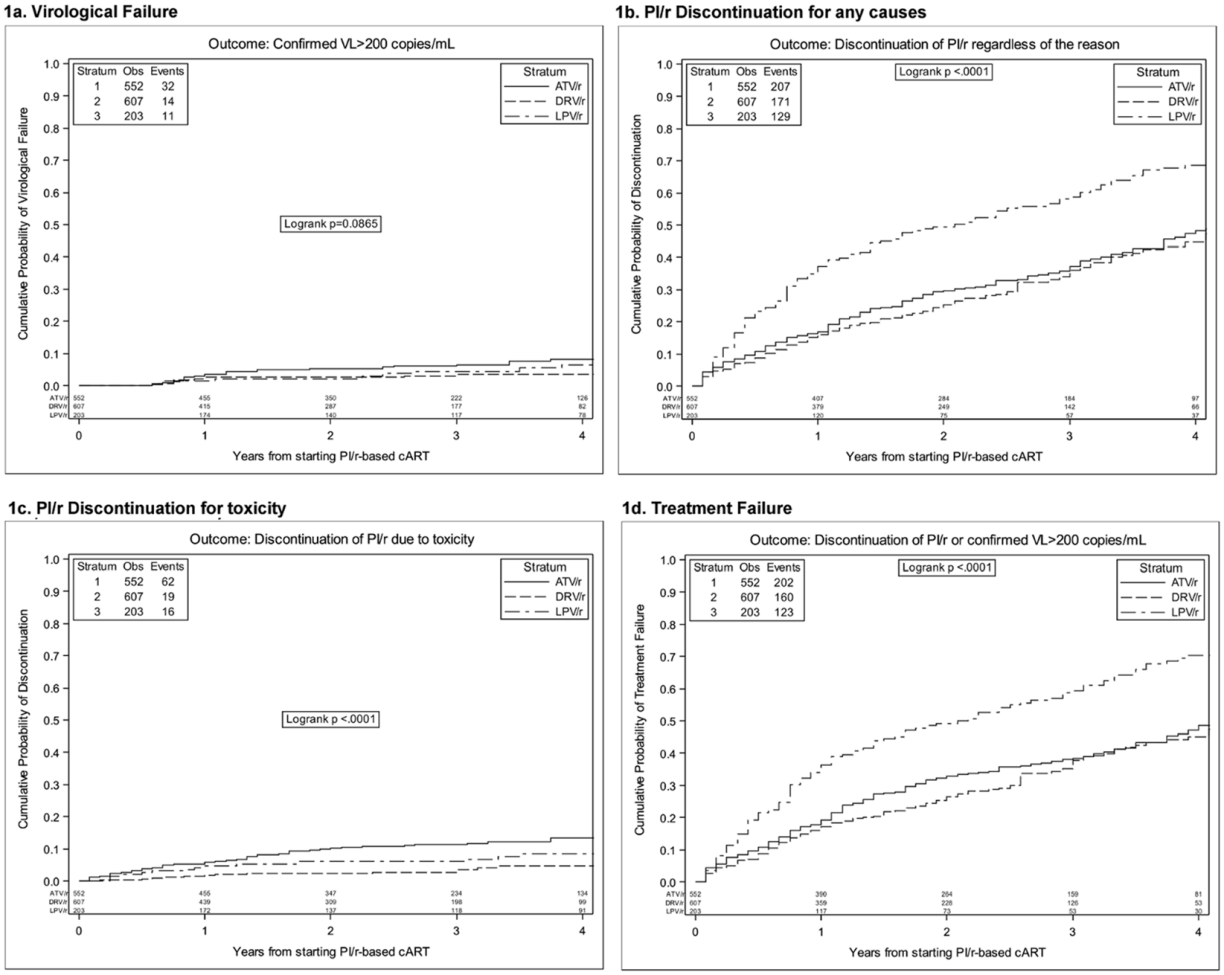
Kaplan Meier curves of the probability of reaching the different end-points according to the PI/r component of the initial cART regimen in 1362 HIV positive LC patients.

The Kaplan Meier (KM) curves of reaching each of the end-points are represented in Fig 1a–1d.

Looking in detail at the different end-points, the KM estimate of VF was 2.8% (95%CI: 1.9–3.8) at 1 year and 5.0% (95%CI: 3.6–6.4) at 3 years; there were no differences in the probability of VF according to the PI/r of the regimen (Fig 1a; log rank test p =.0865). The KM estimate of TD was 19.7% (95%CI: 17.5–22.0) at 1 year and 38.7% (95%CI: 35.6–41.7) at 3 years; the probability of TD was significantly higher for LPV/r-containing regimens in respect of the other two PIs (Fig 1b; log rank test p <.0001). Looking at the probability of discontinuation of the PI/r component of the regimen due to toxicity (Fig 1c) there were disparities according to the PI/r regimens, with DRV/r showing the lower probability of this event and ATV/r the highest (log rank test: p <.0001). Finally, the KM estimate of composite end-point of TF including the probability of either VF or TD was 21.1% (95%CI: 18.7–23.4) at 1 year and 40.3% (95%CI: 37.1–43.4) at 3 years; LPV/r containing regimen showed the worse one (Fig 1d; log rank test: p <.0001)-

In the multivariable Cox model (Table 3), taking into account the possible confounders (age, gender, nation of birth, mode of HIV transmission, hepatitis co-infection status, AIDS diagnosis, nucleoside pair started, baseline CD4 count and viral load and year of starting cART, and stratified by clinical center), there were no differences in the risk of VF according to the regimen used. The adjusted risk of TD was statistically higher for LPV/r including regimens: in comparison to LPV/r-, ATV/r had 53% lower chance and DRV/r 61% lower chance to discontinue the PI component of the regimen. Looking at the risk of TDT, compared to LPV/r, ATV/r regimens had a non significantly 71% higher chance (p = .09) and DRV/r showed a non significantly 49% lower chance (p = .081) of discontinuing the PI because of toxicity. Finally, the chance of TF was significantly lower for both ATV/r (by 51%) and for DRV/r (by 62%) as compared to LPV/r. The same results on different end-points were obtained by fitting the Cox model after excluding patients given DRV/r as bid (data not shown).

**Table 3:** LC patients: RH of various endpoints from fitting a Cox regression analysis.

	Crude and adjusted relative hazards			
	Crude RH (95% CI)	p-value	Adjusted* RH (95% CI)	p-value
***VL>200 copies/mL***				
***Group***				
LPV/r	1.00		1.00	
ATV/r	1.97 (1.11, 3.51)	0.021	1.92 (0.86, 4.28)	0.111
DRV/r	0.63 (0.32, 1.25)	0.185	1.13 (0.45, 2.85)	0.801
***Discontinuation***				
***Group***				
LPV/r	1.00		1.00	
ATV/r	0.51 (0.40, 0.64)	<.001	0.47 (0.37, 0.60)	<.001
DRV/r	0.47 (0.37, 0.59)	<.001	0.36 (0.28, 0.47)	<.001
***Discontinuation due to toxicity***				
***Group***				
LPV/r	1.00		1.00	
ATV/r	1.97 (1.11, 3.51)	0.021	1.71 (0.91, 3.23)	0.095
DRV/r	0.63 (0.32, 1.25)	0.185	0.51 (0.24, 1.09)	0.081
***VL>200 copies/mL or discontinuation***				
***Group***				
LPV/r	1.00		1.00	
ATV/r	0.54 (0.42, 0.67)	<.001	0.49 (0.39, 0.63)	<.001
DRV/r	0.46 (0.36, 0.58)	<.001	0.38 (0.29, 0.50)	<.001

*adjusted for age, gender, nation of birth, mode of HIV transmission,hepatitis co-infection status, AIDS diagnosis, nucleoside pair started,baseline CD4 count and viral load and year of starting cARTand stratified by clinical center

When only the 813 VLC patients were included, in a median follow-up of 15 (6–33) months, 28 patients underwent VF, 162 discontinued the PI/r of their regimen, 28 discontinued because of toxicity and TF occurred in 167 patients. The 1 year-probability of these events was 4.3 (2.7–5.9)% for VF, 22.4 (19.4–25.5)% for TD, 3.9 (2.5–5.4)% for TDT, and 24.2 (21.0–27.4)% for TF.

In these VLC patients, risk of VF was significantly higher for those receiving ATV/r including regimens as compared to LPV/r, in the multivariate model. Similar to the findings among the LC group, both ATV/r and DRV/r showed a lower chance to discontinue the PI component of the regimen as compared to LPV/r including regimens.

DRV/r showed a significantly 69% lower chance (p = .081) of discontinuing the PI because of toxicity as compared to LPV/r. Finally, the chance of TF was significantly lower for both ATV/r (by 44%) and DRV/r (by 66%) as compared to LPV/r (Table 4).

**Table 4:** VLC patients: RH of various endpoints from fitting a Cox regression analysis.

	Crude and adjusted relative hazards			
Outcomes	Crude RH (95% CI)	p-value	Adjusted* RH (95% CI)	p-value
***VL>200 copies/mL***				
***Group***				
LPV/r	1.00		1.00	
ATV/r	1.62 (0.83, 3.18)	0.159	3.70 (1.16, 11.74)	0.027
DRV/r	0.45 (0.20, 1.01)	0.054	3.10 (0.89, 10.80)	0.076
***Discontinuation***				
***Group***				
LPV/r	1.00		1.00	
ATV/r	0.57 (0.43, 0.76)	<.001	0.50 (0.37, 0.69)	<.001
DRV/r	0.41 (0.31, 0.55)	<.001	0.31 (0.22, 0.43)	<.001
***Discontinuation due to toxicity***				
***Group***				
LPV/r	1.00		1.00	
ATV/r	1.62 (0.83, 3.18)	0.159	1.11 (0.51, 2.43)	0.795
DRV/r	0.45 (0.20, 1.01)	0.054	0.31 (0.12, 0.78)	0.013
***VL>200 copies/mL or discontinuation***				
***Group***				
LPV/r	1.00		1.00	
ATV/r	0.64 (0.48, 0.86)	0.003	0.56 (0.41, 0.79)	<.001
DRV/r	0.42 (0.31, 0.57)	<.001	0.34 (0.24, 0.48)	<.001

*adjusted for age, gender, nation of birth, mode of HIV transmission,hepatitis co-infection status, AIDS diagnosis, nucleoside pair started,baseline CD4 count and viral load and year of starting cARTand stratified by clinical center

## Discussion

In our cohort including only severely immunodepressed patients given a first line PI/r containing therapy, we were able to demonstrate several differences in risk of virological failure, tolerability and durability of the different regimens. But first of all, also in these categories of severely immunodepressed patients, and roughly 50% with a viral load >5 log10 HIV-RNA copies/ml, the likehood of virological failure was low, accounting for 2.8% and 4.3% by the first year in those presenting with CD4 counts less than 350 and less than 200/mm^3^ or AIDS respectively. Further, the probability of discontinuation of the PI/r component of the regimen is of 19.7% by one year, similar to what already published on the Icona cohort [15].

In this perspective, we did not found any differences in adjusted risk of virological failure according to the PI/r given in those patients starting cART with CD4 counts less than or equal to 350 cells/mm^3^ or AIDS. Nevertheless, looking at more advanced patients, with CD4 counts up to 200 cells/mm^3^ or AIDS, LPV/r including regimens resulted to be at lower risk of virological failure as in the multivariate model, ATV/r showed a 3.7-fold statistically significant higher risk and DRV/r a 3.1-fold not significant higher risk.

Looking at the other endpoints, i.e. PI/r discontinuation for any causes or for toxicity and treatment failure, we did not find different predictors according to the severity of immune depression: the risk of discontinuing the PI/r of the regimen for any causes was higher for patients given LPV/r as compared the other two PI/r, after adjustment for other possible predictors; limiting the end-point to those discontinuing only because of toxicity, there were no differences in risk of this event when comparing ATV/r to LPV/r, while patients given DRV/r including regimen were at lower risk of discontinuation for toxicity. Finally, LPV/r including regimens were at higher risk of treatment failure as compared to the other PI/r regimens, in both the groups of immune depressed patients.

To date there is no single trial comparing the different PI/r regimens in the population of severely immune depressed patients. Actually, the head to head Castle trial [11] comparing ATV/r vs LPV/r regimens, included patients with median CD4 count of 205, ranging from 2 to 810, cells/cmm, and was powered to demonstrate virological non inferiority between the two regimens in a ITT analysis. A post-hoc analysis demonstrated that lower responses rates were associated with lower baseline CD4 cell counts for the LPV/r but not for the ATV/r group [11].

The Artemis trial [12] was designed to demonstrate non inferiority of DRV/r 800/100 mg once daily vs LPV/r 800/200 mg bid in the proportion of patients with HIV-RNA<50 copies/ml by week 48. The median CD4 counts of the patients was of 228 (4–750) cells/cmm in the DRV/r arm and 218 (2–714) in the LPV/r arm; a total of 42% of patients had CD4 counts<200/cmm at baseline. The trial demonstrated superiority in virologic response in patients taken DRV/r as compared to LPV/r. Patients on DRV/r including regimens discontinued less frequently the regimen because of side effects as compared to those on LPV/r.

Finally the ACTG5257 trial [13] included two PI/r regimens, ATV/r and DRV/r and one integrase including regimen, Raltegravir (RAL) in a open label, randomised, 1:1.1 trial enrolling 1,809 participants, powered to demonstrate equivalence regarding virologic efficacy and tolerability over 96 weeks. The median baseline CD4 count was of 309 cells/cmm; 41% of the population had baseline CD4 counts >350/cmm. Over 2 years the three regimens attained high and equivalent rates of virologic control, but ATV/r including regimens resulted in a higher discontinuation due to tolerability as compared to the other two groups.

Taken together, we can argue that overall there was no major difference in virological potency across the three PI/r in randomized controlled trials, but in case of patients with CD4 counts <200/mm^3^, LPV/r including regimen were less virologically potent than ATV/r ones. This last finding is not confirmed by our data in an observational setting, highlighting that LPV/r including regimens resulted the more potent than both ATV/r and DRV/r (even if in this last case not significantly different) in subjects with very low (< = 200/cmm) CD4 counts or AIDS.

Observational data may be biased for many reasons, but they are more adherent to real life than clinical trials. Possibly, LPV/r including regimens, to be administered twice a day and with a double boosted ritonavir, could be more potent that the other PI/r, as forgiveness could be less important.

Looking at discontinuation, LPV/r including regimens resulted to be those associated to higher risk of discontinuation across both CD4 strata, confirming the data of trials [11–12], particularly linked to the gastrointestinal complains of the double dose of ritonavir.

Also, the higher risk of discontinuing the PI component of the regimen was true also for ATV/r as compared to DRV/r, confirming the finding of the ACTG5257 trial [13], possibly due to hyperbilirubinemia as driver of willingness to discontinue the drug.

It is not surprising that, taking into account both efficacy, tolerability, scheduled timing of administration and toxicities, LPV/r including regimens are those associated to higher risk of treatment failure, accordingly to clinical trails, and actually all guidelines have downgraded LPV/r to a second-line choice in initial cART [4–8], even if it shows a good virological outcome also in the real life setting.

In conclusion, based on our data in a real life setting, focused on severely immunodepressed patients, the overall efficacy and durability of the PI/r including regimens is high, with a likehood of only 4% and 21% of virological failure and discontinuation by one year.

New integrase including regimens, demonstrated to be at least at similar virological potency, but with less toxicities than PI/r in the clinical trials [13, 16–17] should be compared to PI/r including regimens in this setting of advanced patients in order to hopefully further improve potency and durability of antiretroviral therapy.

## Supporting Information

S1 Dataset (XLSX)

S1 Table (DOCX)
