# Supplementary material for: Response to First-Line Ritonavir-Boosted Protease Inhibitors (PI/r)-Based Regimens in HIV Positive Patients Presenting to Care with Low CD4 Counts: Data from the Icona Foundation Cohort
Source: PLoS One. 2016 Jun 27;11(6):e0156360. doi: 10.1371/journal.pone.0156360 (PMC4922579; doi:10.1371/journal.pone.0156360)
Supplement: S1 Table — (DOCX) [file pone.0156360.s002.docx]

Table 1 c

|  | **PI/r started** | | | | |
| --- | --- | --- | --- | --- | --- |
| **Characteristics** | **DRV/r** | **ATV/r** | **LPV/r** | **p-value^*^** | **Total** |
|  | **N= 607** | **N= 552** | **N= 203** |  | **N= 1362** |
| ***Gender, n(%)*** |  |  |  | **0.021** |  |
| **Female** | **134 (22.1%)** | **128 (23.2%)** | **64 (31.5%)** |  | **326 (23.9%)** |
| ***Mode of HIV Transmission, n(%)*** |  |  |  | **0.002** |  |
| **IDU** | **42 (7.0%)** | **64 (11.7%)** | **22 (10.8%)** |  | **128 (9.5%)** |
| **Homosexual contacts** | **230 (38.2%)** | **182 (33.2%)** | **53 (26.1%)** |  | **465 (34.3%)** |
| **Heterosexual contacts** | **278 (45.8%)** | **271 (49.1%)** | **107 (52.7%)** |  | **656 (48.2%)** |
| **Other/Unknown** | **52 (8.6%)** | **32 (5.8%)** | **21 (10.3%)** |  | **105 (7.8%)** |
| ***Nationality, n(%)*** |  |  |  | **0.002** |  |
| **Not Italian** | **151 (24.9%)** | **133 (24.1%)** | **57 (28.1%)** |  | **341 (25.0%)** |
| ***AIDS diagnosis, n(%)*** |  |  |  | **<.001** |  |
| **Yes** | **88 (14.5%)** | **36 (6.5%)** | **26 (12.8%)** |  | **150 (11.0%)** |
| ***CVD diagnosis, n(%)*** |  |  |  | **0.114** |  |
| **Yes** | **7 (1.2%)** | **2 (0.4%)** | **0 (0.0%)** |  | **9 (0.7%)** |
| ***HBsAg, n(%)*** |  |  |  | **0.638** |  |
| **Negative** | **515 (84.8%)** | **462 (83.7%)** | **178 (87.7%)** |  | **1155 (84.8%)** |
| **Positive** | **10 (1.6%)** | **13 (2.4%)** | **4 (2.0%)** |  | **27 (2.0%)** |
| **Not tested** | **82 (13.5%)** | **77 (13.9%)** | **21 (10.3%)** |  | **180 (13.2%)** |
| ***HCVAb, n(%)*** |  |  |  | **0.115** |  |
| **Negative** | **486 (80.1%)** | **434 (78.6%)** | **165 (81.3%)** |  | **1085 (79.7%)** |
| **Positive** | **52 (8.6%)** | **70 (12.7%)** | **21 (10.3%)** |  | **143 (10.5%)** |
| **Not tested** | **69 (11.4%)** | **48 (8.7%)** | **17 (8.4%)** |  | **134 (9.8%)** |
| ***Hepatitis co-infection^*^, n(%)*** |  |  |  | **0.077** |  |
| **No** | **453 (74.6%)** | **393 (71.2%)** | **157 (77.3%)** |  | **1003 (73.6%)** |
| **Yes** | **62 (10.2%)** | **83 (15.0%)** | **24 (11.8%)** |  | **169 (12.4%)** |
| **Not tested** | **92 (15.2%)** | **76 (13.8%)** | **22 (10.8%)** |  | **190 (14.0%)** |
| ***Calendar year of baseline^**^*** |  |  |  | **<.001** |  |
| **Median (IQR)** | **2012 (2011, 2014)** | **2012 (2011, 2013)** | **2011 (2009, 2012)** |  | **2012 (2011, 2013)** |
| ***Age, years*** |  |  |  | **0.119** |  |
| **Median (IQR)** | **40 (33, 49)** | **39 (32, 47)** | **39 (33, 47)** |  | **39 (33, 47)** |
| ***CD4 count nadir, cells/mmc*** |  |  |  | **<.001** |  |
| **Median (IQR)** | **131 (42, 248)** | **212 (99, 285)** | **150 (58, 246)** |  | **165 (59, 266)** |
| ***CD8 count, cells/mmc*** |  |  |  | **0.020** |  |
| **Median (IQR)** | **675 (441, 1001)** | **739 (514, 1151)** | **758 (513, 1147)** |  | **708 (483, 1068)** |
| ***Viral load at first cART, log10 copies/mL*** |  |  |  | **0.036** |  |
| **Median (IQR)** | **5.07 (4.43, 5.55)** | **4.89 (4.26, 5.42)** | **4.99 (4.47, 5.46)** |  | **5.00 (4.40, 5.49)** |
| ***Site geographical position, n(%)*** |  |  |  | **<.001** |  |
| **North** | **310 (51.1%)** | **336 (60.9%)** | **99 (48.8%)** |  | **745 (54.7%)** |
| **Center** | **203 (33.4%)** | **174 (31.5%)** | **74 (36.5%)** |  | **451 (33.1%)** |
| **South** | **94 (15.5%)** | **42 (7.6%)** | **30 (14.8%)** |  | **166 (12.2%)** |
| ***Diabetes, n(%)*** |  |  |  | **0.049** |  |
| **Yes** | **9 (1.5%)** | **14 (2.5%)** | **0 (0.0%)** |  | **23 (1.7%)** |
| ***Smoking, n(%)*** |  |  |  | **0.020** |  |
| **No** | **293 (48.3%)** | **240 (43.5%)** | **103 (50.7%)** |  | **636 (46.7%)** |
| **Yes** | **182 (30.0%)** | **213 (38.6%)** | **61 (30.0%)** |  | **456 (33.5%)** |
| **Unknown** | **132 (21.7%)** | **99 (17.9%)** | **39 (19.2%)** |  | **270 (19.8%)** |
| ***Total cholesterol, mg/dL*** |  |  |  | **0.029** |  |
| **Median (IQR)** | **151 (123, 180)** | **157 (134, 182)** | **160 (140, 184)** |  | **155 (131, 182)** |
| ***HDL cholesterol, mg/dL*** |  |  |  | **0.009** |  |
| **Median (IQR)** | **36 (28, 44)** | **37 (31, 46)** | **37 (30, 47)** |  | **36 (30, 45)** |
| ***Use of statins, n(%)*** |  |  |  | **0.601** |  |
| **Yes** | **7 (1.2%)** | **4 (0.7%)** | **1 (0.5%)** |  | **12 (0.9%)** |
| ***Use of blood pressure lowering drugs, n(%)*** |  |  |  | **0.819** |  |
| **Yes** | **20 (3.3%)** | **21 (3.8%)** | **6 (3.0%)** |  | **47 (3.5%)** |
| ***Time from HIV diagnosis to date of starting cART, months*** |  |  |  | **0.023** |  |
| **Median (IQR)** | **1 (1, 5)** | **2 (1, 25)** | **2 (1, 17)** |  | **2 (1, 13)** |
| ***egfr (CKD_Epi formula), ml/min/1.73m^2^*** |  |  |  | **0.448** |  |
| **Median (IQR)** | **106.7 (93.27, 117.3)** | **107.2 (96.61, 116.4)** | **107.7 (93.30, 117.8)** |  | **107.1 (94.61, 116.8)** |
| ***Blood glucose, mg/dL*** |  |  |  | **0.262** |  |
| **Median (IQR)** | **86 (79, 95)** | **87 (80, 94)** | **86 (79, 94)** |  | **86 (79, 94)** |
| ***NNRTI pair started, n(%)*** |  |  |  | **0.964** |  |
| **Tenofovir/Emtricitabine** | **537 (88.5%)** | **489 (88.6%)** | **181 (89.2%)** |  | **1207 (88.6%)** |
| **Abacavir//Lamivudine** | **70 (11.5%)** | **63 (11.4%)** | **22 (10.8%)** |  | **155 (11.4%)** |
| ***DRV dosage, n(%)*** |  |  |  |  |  |
| **BID** | **87 (14.3%)** |  |  |  |  |
| **QD** | **489 (80.6%)** |  |  |  |  |
| **Unknown** | **31 (5.1%)** |  |  |  |  |
| ***Follow-up, months*** |  |  |  | **<.001** |  |
| **Median (IQR)** | **17 (6, 32)** | **22 (9, 38)** | **15 (5, 37)** |  | **18 (7, 35)** |
| **^*^Chi-square or F-Fisher ANOVA test as appropriate** | | | | | |

Table 1d

|  | **PI/r started** | | | | |
| --- | --- | --- | --- | --- | --- |
| **Characteristics** | **DRV/r** | **ATV/r** | **LPV/r** | **p-value^*^** | **Total** |
|  | **N= 414** | **N= 268** | **N= 131** |  | **N= 813** |
| ***Gender, n(%)*** |  |  |  | **0.037** |  |
| **Female** | **89 (21.5%)** | **59 (22.0%)** | **42 (32.1%)** |  | **190 (23.4%)** |
| ***Mode of HIV Transmission, n(%)*** |  |  |  | **0.002** |  |
| **IDU** | **22 (5.3%)** | **34 (12.8%)** | **12 (9.2%)** |  | **68 (8.4%)** |
| **Homosexual contacts** | **140 (34.0%)** | **71 (26.7%)** | **30 (22.9%)** |  | **241 (29.8%)** |
| **Heterosexual contacts** | **207 (50.0%)** | **143 (53.4%)** | **73 (55.7%)** |  | **423 (52.0%)** |
| **Other/Unknown** | **43 (10.4%)** | **18 (6.8%)** | **16 (12.2%)** |  | **77 (9.5%)** |
| ***Nationality, n(%)*** |  |  |  | **0.001** |  |
| **Not Italian** | **112 (27.1%)** | **69 (25.7%)** | **42 (32.1%)** |  | **223 (27.4%)** |
| ***AIDS diagnosis, n(%)*** |  |  |  | **0.033** |  |
| **Yes** | **88 (21.3%)** | **36 (13.4%)** | **26 (19.8%)** |  | **150 (18.5%)** |
| ***CVD diagnosis, n(%)*** |  |  |  | **0.387** |  |
| **Yes** | **4 (1.0%)** | **1 (0.4%)** | **0 (0.0%)** |  | **5 (0.6%)** |
| ***HBsAg, n(%)*** |  |  |  | **0.463** |  |
| **Negative** | **353 (85.3%)** | **226 (84.3%)** | **117 (89.3%)** |  | **696 (85.6%)** |
| **Positive** | **7 (1.7%)** | **8 (3.0%)** | **1 (0.8%)** |  | **16 (2.0%)** |
| **Not tested** | **54 (13.0%)** | **34 (12.7%)** | **13 (9.9%)** |  | **101 (12.4%)** |
| ***HCVAb, n(%)*** |  |  |  | **0.066** |  |
| **Negative** | **334 (80.7%)** | **209 (78.0%)** | **109 (83.2%)** |  | **652 (80.2%)** |
| **Positive** | **31 (7.5%)** | **36 (13.4%)** | **10 (7.6%)** |  | **77 (9.5%)** |
| **Not tested** | **49 (11.8%)** | **23 (8.6%)** | **12 (9.2%)** |  | **84 (10.3%)** |
| ***Hepatitis co-infection^*^, n(%)*** |  |  |  | **0.020** |  |
| **No** | **314 (75.8%)** | **192 (71.6%)** | **106 (80.9%)** |  | **612 (75.3%)** |
| **Yes** | **38 (9.2%)** | **44 (16.4%)** | **11 (8.4%)** |  | **93 (11.4%)** |
| **Not tested** | **62 (15.0%)** | **32 (11.9%)** | **14 (10.7%)** |  | **108 (13.3%)** |
| ***Calendar year of baseline^**^*** |  |  |  | **<.001** |  |
| **Median (IQR)** | **2012 (2011, 2014)** | **2012 (2011, 2013)** | **2011 (2009, 2012)** |  | **2012 (2011, 2013)** |
| ***Age, years*** |  |  |  | **0.986** |  |
| **Median (IQR)** | **41 (34, 50)** | **41 (34, 50)** | **41 (34, 49)** |  | **41 (34, 50)** |
| ***CD4 count nadir, cells/mmc*** |  |  |  | **0.107** |  |
| **Median (IQR)** | **68 (29, 132)** | **99 (40, 153)** | **85 (33, 150)** |  | **78 (32, 143)** |
| ***CD8 count, cells/mmc*** |  |  |  | **0.143** |  |
| **Median (IQR)** | **561 (343, 842)** | **609 (408, 955)** | **655 (370, 1047)** |  | **593 (370, 906)** |
| ***Viral load at first cART, log10 copies/mL*** |  |  |  | **0.595** |  |
| **Median (IQR)** | **5.26 (4.65, 5.72)** | **5.18 (4.60, 5.60)** | **5.09 (4.57, 5.63)** |  | **5.20 (4.61, 5.67)** |
| ***Site geographical position, n(%)*** |  |  |  | **0.136** |  |
| **North** | **207 (50.0%)** | **149 (55.6%)** | **61 (46.6%)** |  | **417 (51.3%)** |
| **Center** | **140 (33.8%)** | **91 (34.0%)** | **53 (40.5%)** |  | **284 (34.9%)** |
| **South** | **67 (16.2%)** | **28 (10.4%)** | **17 (13.0%)** |  | **112 (13.8%)** |
| ***Diabetes, n(%)*** |  |  |  | **0.084** |  |
| **Yes** | **8 (1.9%)** | **9 (3.4%)** | **0 (0.0%)** |  | **17 (2.1%)** |
| ***Smoking, n(%)*** |  |  |  | **0.282** |  |
| **No** | **201 (48.6%)** | **120 (44.8%)** | **64 (48.9%)** |  | **385 (47.4%)** |
| **Yes** | **116 (28.0%)** | **96 (35.8%)** | **39 (29.8%)** |  | **251 (30.9%)** |
| **Unknown** | **97 (23.4%)** | **52 (19.4%)** | **28 (21.4%)** |  | **177 (21.8%)** |
| ***Total cholesterol, mg/dL*** |  |  |  | **0.009** |  |
| **Median (IQR)** | **143 (117, 173)** | **153 (130, 177)** | **164 (138, 183)** |  | **149 (125, 177)** |
| ***HDL cholesterol, mg/dL*** |  |  |  | **0.082** |  |
| **Median (IQR)** | **33 (26, 42)** | **36 (29, 45)** | **35 (28, 43)** |  | **34 (27, 43)** |
| ***Use of statins, n(%)*** |  |  |  | **0.805** |  |
| **Yes** | **5 (1.2%)** | **2 (0.7%)** | **1 (0.8%)** |  | **8 (1.0%)** |
| ***Use of blood pressure lowering drugs, n(%)*** |  |  |  | **0.711** |  |
| **Yes** | **14 (3.4%)** | **8 (3.0%)** | **6 (4.6%)** |  | **28 (3.4%)** |
| ***Time from HIV diagnosis to date of starting cART, months*** |  |  |  | **0.044** |  |
| **Median (IQR)** | **1 (0, 2)** | **1 (0, 2)** | **1 (0, 5)** |  | **1 (0, 2)** |
| ***egfr (CKD_Epi formula), ml/min/1.73m^2^*** |  |  |  | **0.448** |  |
| **Median (IQR)** | **108.5 (93.07, 117.8)** | **107.9 (95.00, 115.8)** | **107.7 (93.87, 117.3)** |  | **108.0 (93.82, 116.8)** |
| ***Blood glucose, mg/dL*** |  |  |  | **0.405** |  |
| **Median (IQR)** | **86 (79, 96)** | **88 (80, 96)** | **87 (78, 97)** |  | **87 (79, 96)** |
| ***NNRTI pair started, n(%)*** |  |  |  | **0.593** |  |
| **Tenofovir/Emtricitabine** | **371 (89.6%)** | **246 (91.8%)** | **117 (89.3%)** |  | **734 (90.3%)** |
| **Abacavir//Lamivudine** | **43 (10.4%)** | **22 (8.2%)** | **14 (10.7%)** |  | **79 (9.7%)** |
| ***DRV dosage, n(%)*** |  |  |  |  |  |
| **BID** | **61 (14.7%)** |  |  |  |  |
| **QD** | **327 (79.0%)** |  |  |  |  |
| **Unknown** | **26 (6.3%)** |  |  |  |  |
| ***Follow-up, months*** |  |  |  | **0.088** |  |
| **Median (IQR)** | **16 (6, 31)** | **17 (7, 35)** | **13 (5, 34)** |  | **15 (6, 33)** |
| **^*^Chi-square or F-Fisher ANOVA test as appropriate** | | | | | |
